# Transcriptomic Analysis Reveals Key Genes Related to Betalain Biosynthesis in Pulp Coloration of *Hylocereus polyrhizus*

**DOI:** 10.3389/fpls.2015.01179

**Published:** 2016-01-05

**Authors:** Hua Qingzhu, Chen Chengjie, Chen Zhe, Chen Pengkun, Ma Yuewen, Wu Jingyu, Zheng Jian, Hu Guibing, Zhao Jietang, Qin Yonghua

**Affiliations:** State Key Laboratory for Conservation and Utilization of Subtropical Agro-Bioresources/Key Laboratory of Biology and Genetic Improvement of Horticultural Crops-South China, Ministry of Agriculture, College of Horticulture, South China Agricultural UniversityGuangzhou, China

**Keywords:** *Hylocereus polyrhizus*, transcriptome, RNA-Seq, betalain biosynthesis, expression analyses

## Abstract

Betalains have high nutritional value and bioactivities. Red pulp pitaya (*Hylocereus polyrhizus*) is the only fruit containing abundant betalains for consumer. However, no information is available about genes involved in betalain biosynthesis in *H. polyrhizus*. Herein, two cDNA libraries of pitaya pulps with two different coloration stages (white and red pulp stages) of Guanhuahong (*H. polyrhizus*) were constructed. A total of about 12 Gb raw RNA-Seq data was generated and was *de novo* assembled into 122,677 transcripts with an average length of 1183 bp and an N50 value of 2008. Approximately 99.99% of all transcripts were annotated based on seven public databases. A total of 8871 transcripts were significantly regulated. Thirty-three candidate transcripts related to betalain biosynthesis were obtained from the transcriptome data. Transcripts encoding enzymes involved in betalain biosynthesis were analyzed using RT-qPCR at the whole pulp coloration stages of *H. polyrhizus* (7-1) and *H. undatus* (132-4). Nine key transcripts of betalain biosynthesis were identified. They were assigned to four kinds of genes in betalain biosynthetic pathway, including tyrosinase, 4, 5-DOPA dioxygenase extradiol, cytochrome P450 and glucosyltransferase. Ultimately, a preliminary betalain biosynthetic pathway for pitaya was proposed based on betalain analyses, gene expression profiles and published documents.

## Introduction

Plant pigments are classified into four categories i.e., chlorophyll, carotenoid, anthocyanin, and betalains. Betalains not only play important roles in ranging in color from red to violet to yellow in plants, but also have high nutritional value and positive effects in health and disease by high antioxidant and anti-inflammatory capabilities (Swarna et al., 2013; Allegra et al., 2014; Lee et al., 2014; Clifford et al., 2015; Martinez et al., 2015). It is interesting that betalains and anthocyanin cannot co-exist naturally in one plant at the same time (Stafford, 1994). In contrast to anthocyanins and carotenoids, the biosynthetic pathway of betalains is only partially understood (Gandia-Herrero et al., 2005a,b; Tanaka et al., 2008). Studies on betalain biosynthesis are mainly focused on characterizations of several key enzymes, such as tyrosinase (TYR), 4, 5-DOPA-dioxygenase (DOD), and glucosyltransferases (GT). Betalain biosynthetic pathways have been determined or inferred based on characterization and activity analyses of those key enzymes (Gandia-Herrero and Garcia-Carmona, 2013).

Betalains are water-soluble nitrogen-containing pigments derived from tyrosine (Gandia-Herrero and Garcia-Carmona, 2013). TYR activities related to betalain biosynthesis are well-understood (Gandia-Herrero et al., 2005a,b; Nakatsuka et al., 2013). The formation of tyr-betaxanthin and dopaxanthin was carried out by TYR (Gandia-Herrero et al., 2005b). TYR catalyzes formations of L-3,4-dihydroxyphenylalanine (L-DOPA) and 5,6-dihydroxyindoline-2- carboxylic acid (*cyclo*-DOPA) which are the pivotal precursors for betalain biosynthesis. Higher tyrosine-hydroxylating activities played a prominent role in betacyanin formation through TYR hydroxylation and oxidation reactions (Strack et al., 2003; Wang et al., 2007). *TYR* has been obtained from *Phytolacca americana, Spinacia oleracea* and *Suaeda salsa*, but its function in betalain biosynthesis is not clear yet (Hind et al., 1995; Joy et al., 1995; Ma et al., 2013). Recently, a novel cytochrome P450 (from tyrosine to cyclo-DOPA) catalyzing *cyclo*-DOPA formation was obtained from *B. vulgaris* (Hatlestad et al., 2012), *Amaranthus hypochondriacus* (Casique-Arroyo et al., 2014) and *Mirabilis jalapa* (Suzuki et al., 2014). DOD catalyzes L-DOPA to form an unstable seco-DOPA intermediate, and then seco-DOPA spontaneously converts to betalamic acid identified as chromophore. DOD has been extensively studied in betalain-producing plant species such as *Amaranthus hypochondriacus* (Casique-Arroyo et al., 2014), *Portulaca grandiflora* (Christinet et al., 2004; Takahashi et al., 2009), *Suaeda salsa* (Ruan, 2008; Zhao et al., 2011), *B. vulgaris* (Gandia-Herrero and Garcia-Carmona, 2012), *Opuntia ficus-indica* (Stintzing et al., 2005), *Mirabilis jalapa* and *Bougainvillea glabra* (Sasaki et al., 2009), and *Parakeelya mirabilis* (Chung et al., 2015).

Transcription factors or regulatory genes are also involved in betalain-producing plants. MYB1 (*BvMYB1*) was verified to regulate betalain pathway in *B. vulgaris* (Hatlestad et al., 2015). Stracke et al. (2014) obtained 70 R2R3-MYB genes as well as genes encoding three other classes of MYB proteins containing multiple MYB repeats from *B. vulgaris*. Those R2R3-MYB genes were functionally categorized which led to the identification of a sugar beet-specific clade with an atypical amino acid composition in the R3 domain, putatively encoding betalain regulators (Stracke et al., 2014).

Glucosyltransferases was the last key enzyme in betalain biosynthetic pathway to keep stability and diversity of generated pigments. However, enzymes involved in the process have not been identified. There are two glycosylated pathways in the betalain biosynthesis. One is the catalyzed reaction at betanidin 5-O and 6-O location, respectively from betanidin-5-O-glucosyltransferase (B5GT) and betanidin-6-O-glucosyltransferase (B6GT). Betanidin glucosyltransferases have been isolated from *Dorotheanthus bellidiformis* (Vogt et al., 1999; Vogt, 2002), *Phytolacca americana* (Noguchi et al., 2009), *B. vulgaris* (Sepulveda-Jimenez et al., 2004; Isayenkova et al., 2006), *Opuntia ficus-indica* (Stintzing et al., 2005), and *Amaranthus hypochondriacus* (Casique-Arroyo et al., 2014). The other is occurred on *cyclo*-DOPA, that cyclo-DOPA 5-O-glucosyltransferase (CDOPA5GT) catalyzes the formation of betanin. The CDOPA5GT activities have also been found in *Amaranthus hypochondriacus* (Casique-Arroyo et al., 2014), *Celosia cristata* and *Mirabilis jalapa* (Sasaki et al., 2005).

Dragon fruit or pitaya is one of the tropical fruits under *Hylocereus* of the Cactaceae. Currently, there are two types of pitayas i.e., red-flesh pitaya (*H. polyrhizus*) and white-flesh pitaya (*H. undatus*) that have been commercially produced by the large-scale as a new type fruit crop. Pitaya has been drawn much attention of the world for its commercial value and excellent nutritional properties (Adnan et al., 2011). Previous studies showed that major pigment compounds of pitaya are water-soluble betalains in term of red-violet betacyanins and yellow/orange betaxanthin (Stintzing et al., 2002). To date, red pulp pitaya (*H. polyrhizus*) is the only fruit containing betalains for consumer. Pitaya pigment has attracted much attention for its antioxidant and antiproliferative activities and become one of the hot research topics in the world. In the past decades, great progress has been made in pitaya betalain in term of physical and chemical properties (Esquivel et al., 2007; Woo et al., 2011), purification and identification (Stintzing et al., 2002; Wybraniec et al., 2009; Naderi et al., 2010; Rebecca et al., 2010a,b; Lim et al., 2011), antioxidant and radical scavenging capacity (Wu et al., 2006; Tenore et al., 2012; Garcia-Cruz et al., 2013). Several tentative betalain biosynthesis-related compounds were identified and compared according to metabolite profiling of red pulp (*H. polyrhizus*) and white pulp (*H. undatus*) pitayas (Suh et al., 2014). However, genes related to betalain biosynthetic pathway in pitaya is not clear yet. RNA-Seq is a technique that allows rapid and comprehensive understanding of transcriptome level of variations based on next-generation sequencing technologies. The application of RNA-seq has accelerated gene expression profiling and gene identification in many plant species. In this study, RNA-Seq technology was used to screen key genes related to betalain biosynthesis in pulp coloration of Guanhuahong (*H. polyrhizus*). The aim of the present study is to isolate key genes encoding steps in betalain biosynthetic pathway in pitaya.

## Materials and methods

### Plant materials

One varieties i.e., Guanhuahong (*H. polyrhizus*) and two superior pitaya selections i.e., 7-1 (*H. polyrhizus*) and 132-4 (*H. undatus*) with excellent quality were used as materials. Plants were cultivated in Dalingshan Forest Park, Dongguan City, Guangdong Province, China. The same pulps from Guanghuahong were collected for RNA-Seq and data validation by RT-qPCR on the 28^th^ (white pulp stage) and 42^nd^ days (red pulp stage) after artificial pollination (DAAP) (Figures S1A,B) in October and November, 2013. The temperature was 16–32°C with relatively dry climate. Two transcriptome libraries were generated by pooling equal quantities of RNA from each of three fruit developmental stages. Each of these libraries consisted of equal amounts of RNA from three biological replicates of each developmental stage. A pool of pulps from three fruits of 7-1 and 132-4 were collected from four different plants for betalain measurement and expression analyses of candidate transcripts related to betalain biosynthesis on the 19^th^, 22^nd^, 23^rd^, 24^th^, 25^th^, 26^th^, 27^th^, and 28^th^ (Figures S1C,D) DAAP in July and August, 2014. The temperature is 24–36°C with relatively humid climate. All samples were immediately frozen in liquid nitrogen and stored at -80°C until use.

### Measurement of betalains

Betalain contents were extracted and measured following the method of Garcia-Cruz et al. (2013) with a minor modification. 0.5 g fresh pulps were ground into fine powder in liquid nitrogen and extracted with 5 mL 80% aqueous methanol (v/v) solution. Samples were sonicated for 10 min in a ultrasonic cleaner (SB25-12DT, Ningbo, China) and then stirred for 20 min in darkness at room temperature. Supernatants were collected at 2200 × g for 10 min and the residues were subjected to a similar second extraction. The supernatants were measured through spectrophotometry (Infinite M200, Tecan Co.). Betacyanin and betaxanthin contents were calculated by the following formulas: betacyanins or betaxanthin contents (mg/100 g fresh pulps) = (A_538_ or A_483_ × DF × W × V × 100)/(ε × P × L). A_538_ is absorbance for betacyanins at 538 nm (max); and A_483_ is absorbance for betaxanthins, DF is a dilution factor, W is the molecular weight (550 g/mol for betanin and 308 g/mol for indicaxanthin); V is the pigment solution volume (ml); ε is the molar extinction coefficient (60,000 L/mol·cm for betanin and 48,000 L/mol·cm for indicaxanthin), and L is the length of the cell (1 cm). P is the fresh pigment weight (g). All determinations were performed in triplicate.

### Experimental procedures of RNA-Seq

#### Total RNA extraction, library construction, and ILLUMINA deep sequencing

Total RNA extraction, library construction and RNA-Seq were performed by Bo'ao Biotechnology Corporation (Beijing, China). RNA samples were extracted using the TruSeq RNA Sample Preparation Kit (Autolab Biotechnology, Beijing) according to the manufacture's protocol. RNA quality and quantity were analyzed by 1% agarose gel and NanoDrop ND1000 spectrophotometer (NanoDrop Technologies, Wilmington, DE, USA). The RNA integrity number (RIN) values (>7.0) were assessed using an Agilent 2100 Bioanalyzer (Santa Clara, CA, USA). Briefly, the poly-A containing mRNA molecules were purified from 3.0 μg total RNA using poly-T oligo-attached magnetic beads. The cleaved RNA fragments were reversely transcribed into the first-strand cDNA using random hexamers, following by the second-strand cDNA synthesis using DNA polymerase I and RNase H. The cDNA fragments were purified, end blunted, “A” tailed, and adaptor ligated. PCR was used to selectively enrich those DNA fragments that have adapter molecules on both ends and to amplify the amount of DNA in the library. The number of PCR cycles was minimized to avoid skewing the representation of the library. The two libraries were qualified by Agilent 2100 bioanalyzer and quantified by Qubit and RT-qPCR. The produced libraries were sequenced on the HiSeq 2500 platform.

### *De novo* transcriptome data processing and assembly

A Perl program (Bo'ao Biotechnology Corporation, Beijing, China) was used to filter out low quality sequences from raw sequencing data. The quality of each base was checked from the first base of each read. Once a low-quality base (quality < 10) was detected, it was removed together with following sequences. For paired-end reads, if one read was less than 30 bases after the trimming of low quality bases, the whole paired-end reads were eliminated. Then the high quality reads (High-Quality ≥ 10, Length Cutoff ≥ 30 bp) were assembled with software trinityrnaseq- r2013-11-10 (Grabherr et al., 2011) to construct unique consensus sequences.

### Functional annotation and classification

Unigenes were compared with the NCBI Non-redundant nucleotide and protein database (NR, Jan, 2013) (http://www.ncbi.nlm.nih.gov/) using BLASTN and BLASTX (Altschul et al., 1997) with the same *E*-value cutoffs ≤ 1e^−5^, respectively. Additionally, unigenes were aligned to a series of protein databases using BLASTx (Altschul et al., 1997) (*E*-value ≤ 1e^−10^) to obtain the annotation and classification by Swiss-Prot (SWISS-PROT downloaded from European Bioinformatics Institute by Jan, 2013) (Altschul and Gish, 1996), Clusters of Orthologous Groups of proteins database (COG) (Tatusov et al., 1997, 2003), Kyoto Encyclopedia of Genes and Genomes database (KEGG, release 58) (Kanehisa et al., 2006), InterProScan (Zdobnov and Apweiler, 2001) and Gene Ontology (GO) (Harris et al., 2004).

### Detection of differentially expressed unigene

The edgeR software (Robinson et al., 2010) was used to identify differentially expressed genes between the two libraries based on the negative binomial distribution by empirical Bayes estimation and exact tests. Genes with a *P*-value ≤ 0.01 and expression ratio ≥ 2 or expression ratio ≤ 0.5 were considered significant difference between the two libraries.

### RT-qPCR validation of differentially expressed transcripts

Some differentially expressed genes were selected to validate the accuracy of RNA-Seq data using RT-qPCR. All betalains-related transcripts were further elucidated their expression patterns at all pulp coloration stages of 7-1 and 132-4. The pitaya *actin* gene was used as the internal control for normalization of gene expression (Yan et al., 2013). The primers of RT-qPCR (Table S1) were designed using BatchPrimer3 v1.0 (http://batchprimer3.bioinformatics.ucdavis.edu/index.html). Total RNA was isolated from pulp the 19^th^, 22^nd^, 23^rd^, 24^th^, 25^th^, 26^th^, 27^th^, and 28^th^ (Figures S1C,D) DAAP pulp from 7-1 and 132-4 using the Quick RNA isolation Kit (0416-50) (Huayueyang Biotechnology, Beijing) according to the manufacture's protocol, respectively. After treated with DNase I (TaKaRa, Japan), single-stranded cDNA was synthesized using the HiScript®II 1st Strand cDNA Synthesis Kit (Vazyme Biotech, Nanjing). RT-qPCR was performed in an Applied Biosystems 7500 real-time PCR system (Applied Biosystems, CA, USA) using the SYBR® Premix Ex *Taq*™ II (Tli RNaseH Plus) (TaKaRa, Japan). Twenty microliters of the qPCR reaction volume contained 10.0 μl SYBR® Premix Ex *Taq*™ II (2^*x*^), 0.4 μl ROX Reference Dye (50 ×), 0.75 μl PCR forward primer and 0.75 μl PCR reverse primer (1.875 μM), 7.1 μl ddH_2_O, and 1.0 μl cDNA (70 ng). The qPCR parameters were: 95°C for 1 min then 40 cycles of 95°C for 15 s, 56°C for 15 s, and 72°C for 40 s. Melting curve and agarose gel electrophoresis analysis were performed to confirm the PCR specificity. All experiments were repeated in technical triplicate. The relative expression levels for each transcript were calculated using the formula 2^−ΔΔ*Ct*^ method.

## Results

### Betacyanin and betaxanthin contents at all pulp coloration stages of pitaya

**Figure 1:**
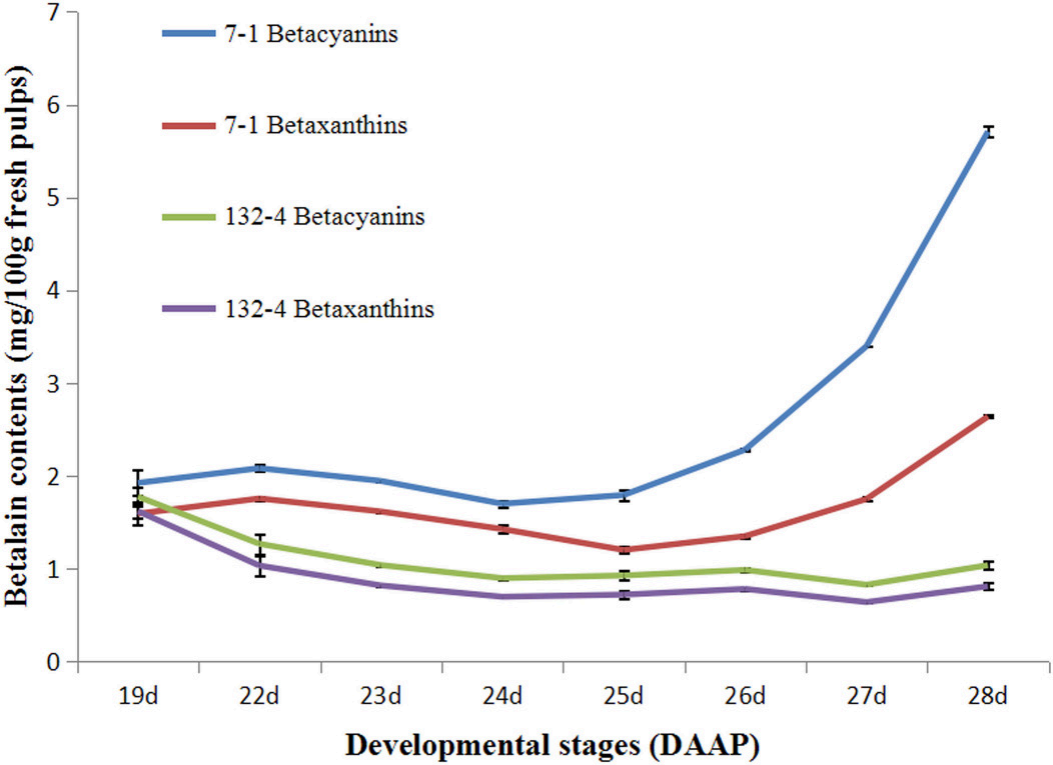
**Contents of betacyanins and betaxanthins at all pulp coloration stages of 7-1 and 132-4. 7-1, *H. polyrhizus*; 132-4, *H. undatus***.

Betalain contents were determined at all stages of pulp coloration in *H. polyrhizus* and *H. undatus*. As shown in Figure 1, higher contents of betacyanin and betaxanthin were detected in *H. polyrhizus* (7-1) than that of *H. undatus* (132-4). Betacyanin and betaxanthin contents were increasing at all stages of pulp coloration in *H. polyrhizus* (7-1) while there were no significant changes in *H. undatus* (132-4) (Figure 1).

### Sequencing and *De novo* assembly of transcriptome

As shown in Table 1, two libraries of the red and white pulp stages produced 67,123,022 and 63,770,742 raw reads (NCBI accessions: SRR2924904), respectively. A single read length was 100 bp. After filtering out low quality reads, 62,639,274 reads (88.71% of the raw data) and 59,004,932 reads (87.33% of the raw data) were obtained, respectively from red and white pulp libraries. Q20 are 96.28 and 95.67%, respectively. The high-quality reads were assembled into 122,677 transcripts with an average length of 1183 bp and an N50 value of 2008 by Trinity software. The range of transcripts length was 201–12,560 bp. The length distribution of these transcripts was shown in Figure S2. The percentages of mapping to transcript were 89.69 and 87.37%, respectively.

**Table 1:** **Summary of the sequencing data of Guanhuahong (*H. polyrhizus*)**.

**Library**	**No. of reads**	**Single length (bp)**	**Paired-end? (Y/N)**	**Total length (bp)**	**Q20**		High quality transcripts^*^		
					**length (bp)**	**%**	**Number**	**Length (bp)**	**%**
Red	33,561,511	100	Y	6,712,302,200	6,462,324,972	96.28	62,639,274	5,954,789,192	88.71
White	31,885,371	100	Y	6,377,074,200	6,100,886,291	95.67	59,004,932	5,569,178,614	87.33

* High-Quality ≥ 10; Length Cutoff ≥ 30 bp.

### Functional annotation and analyses

**Figure 2:**
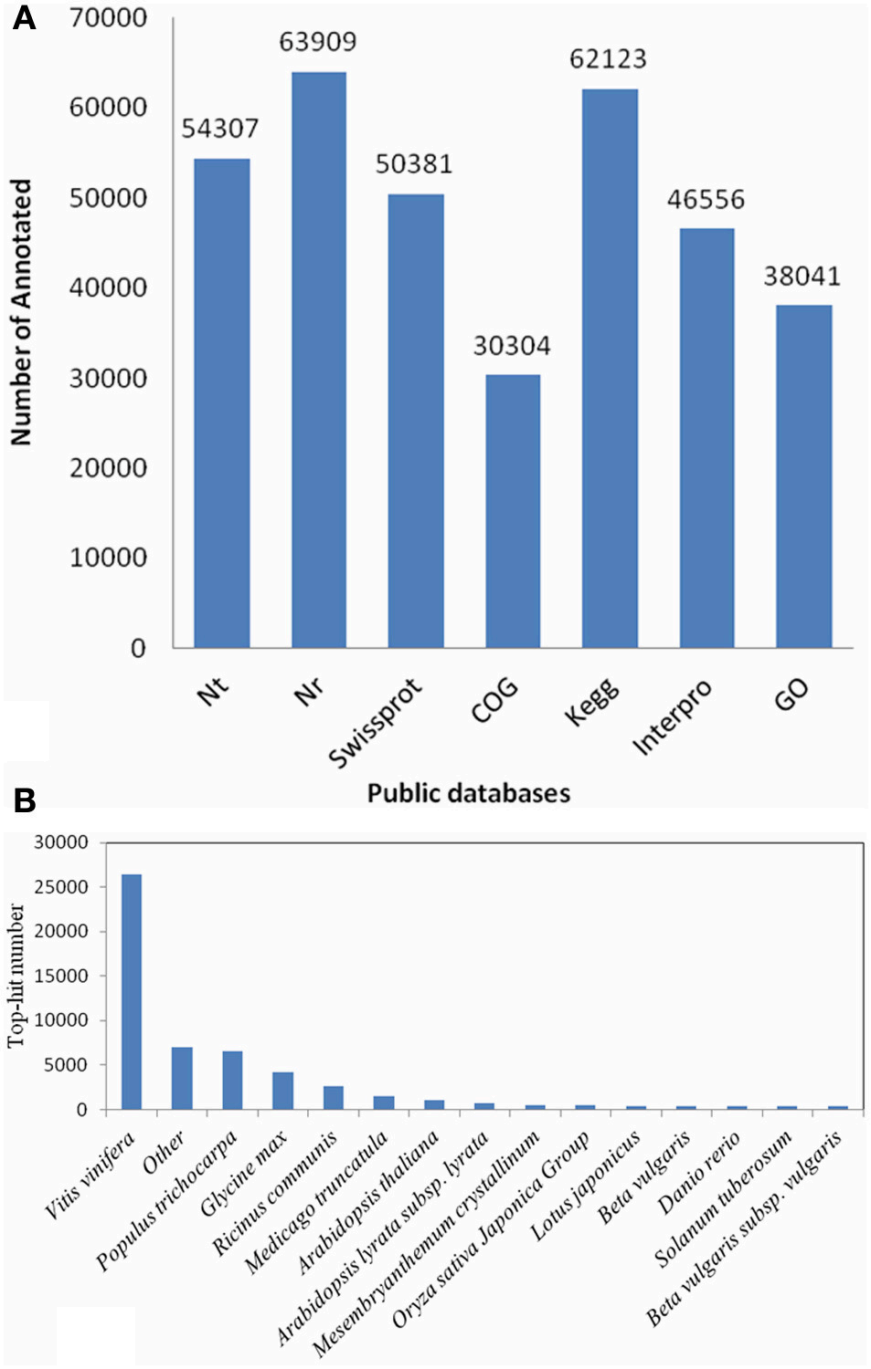
**Functional annotation and distribution of RNA-Seq data. (A)** Sequences statistics of functional annotation for each database. **(B)** Species distribution of the top BLAST hits for total homologous sequences.

Due to lacking of a reference genome in *H. polyrhizus*, 122,677 transcripts were blasted against seven public databases (Nt, Nr, Swiss-Prot, KEGG, COG, GO and Interpro) using search tools. 122,668 (99.99% of all) transcripts were annotated using these databases. The annotation summary was shown in Figure 2A. The species distribution with the greatest number of *H. polyrhizus* were *Vitis vinifera* (50.1%), *Populus trichocarpa* (12.4%), *Glycine max* (7.9%), *Ricinus communis* (4.9%), and *Arabidopsis thaliana* (2.9%) (Figure 2B). Approximately 38,041 transcripts were mainly classified into three categories i.e., cellular component, molecular function and biological process (Figure S3A). The main functions were gathering in “binding” (24,055 transcripts, 63.2%) and “catalytic” (18,882 transcripts, 49.6%) of molecular function classification. As for the biological process, they were focused on “cellular process” (17,101 transcripts, 45%), “metabolic process” (19,353 transcripts, 50.9%) and “pigmentation process” (3298 transcripts, 8.7%). Based on COG classifications, 30,304 transcripts were divided into 24 different functional groups (Figure S3B). 62,123 transcripts were assigned to 309 KEGG pathways (File S1). Sixteen and forty-four transcripts were found respectively in “flavone and flavonol biosynthesis” and “flavonoid biosynthesis.”

### Analysis of differentially expressed transcripts between white and red pulp stages

**Figure 3:**
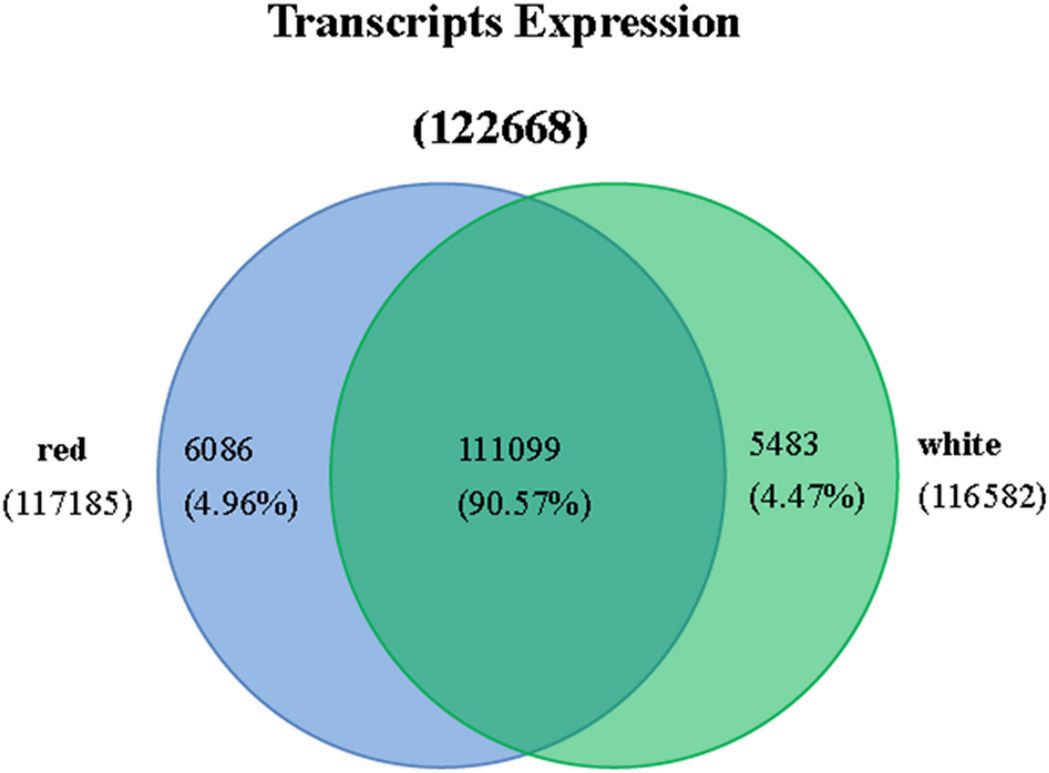
**Expression statistics of transcript from red and white samples of Guanhuahong (*H. polyrhizus*)**.

**Figure 4:**
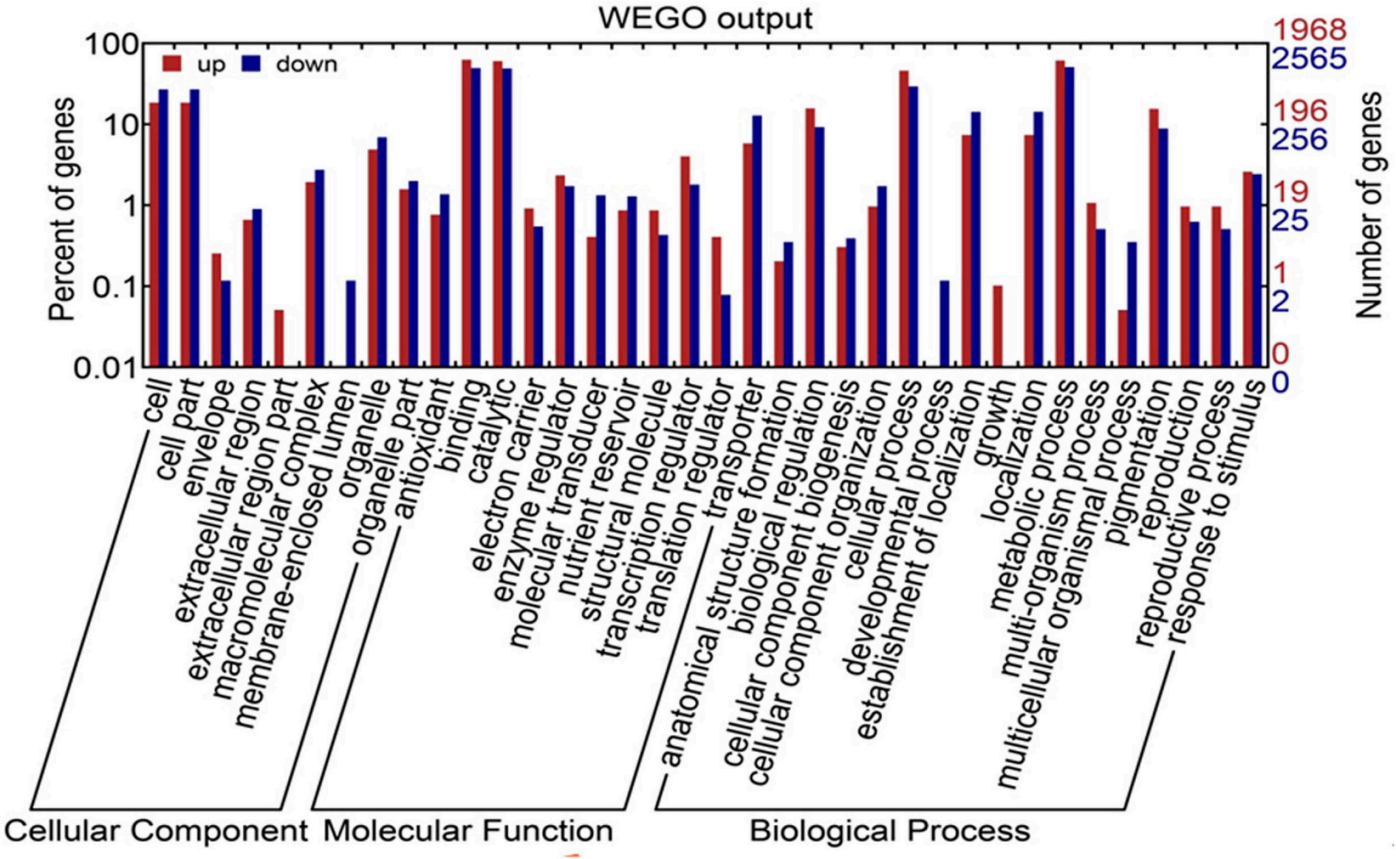
**GO classification of differentially expressed transcripts between white and red samples**. Red strips indicate up-regulated transcripts; blue strips indicate down-regulated transcripts.

As shown in Figure 3 and Table S2, 117,185 transcripts were distributed in red pulp library, and 116,582 were in white pulp library. 6086 and 5483 transcripts independently existed in red and white pulp libraries, respectively. Differentially expressed transcripts were identified by comparisons with the two libraries using *p* ≤ 0.01 and fold changes (ratio ≥ 2 or ratio ≤ 0.5) as thresholds. A total of 8871 transcripts were considered as significantly differentially expressed between the white and red pulp libraries and were assigned to 241 KEGG pathways (File S2). 4107 were up-regulated transcripts. The differentially expressed transcripts were classified into three categories in GO assignments: cellular component, molecular function and biological process (Figure 4). As expected, the differentially expressed transcripts involved in pigmentation were presented in the biological process. In flavone and flavonol biosynthesis (ko00944), differentially expressed transcripts were focused on flavonol 3-O-methyltransferase (E2.1.1.76, up-regulated expression, *comp37084_c6_seq1, comp37084_c6_seq2, comp37385_c0_seq2, comp37385_c0_seq1, comp34873_c0_seq1, comp31547_c0_seq1*) and flavonoid 3′-monnooxygenase comp (E1.14.13.21, down-regulated expression, *comp32441_c0_seq1*). There were 9 up-regulated transcripts and 10 down-regulated transcripts in flavonoid biosynthesis. All up-regulated transcripts were annotated as chalcone isomerase (CHI) and chalcone synthase (CHS). Dihydroflavonol reductase (DFR), anthocyanidin synthase (ANS), and leucoanthocyanidin reductase (LAR) were down-regulated expression genes in pitaya which is consistent with the conjecture of betalain-producing plants (Shimada et al., 2005).

### Genes involved in betalain biosynthesis

**Figure 5:**
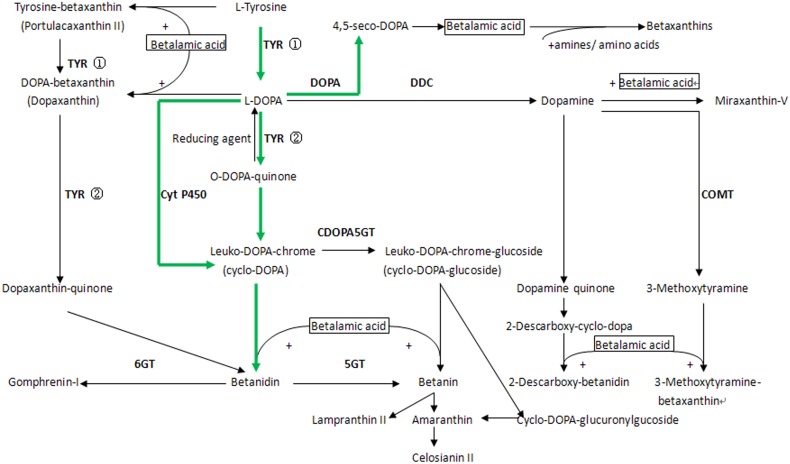
**A diagram of the comprehensive betalains biosynthesis pathway in plant from KEGG pathway and Gandia-Herrero's review (Gandia-Herrero and Garcia-Carmona, 2013)**. A preliminary betalain pathway in *H. polyrhizus* was shown by green arrows. ①, tyrosinase hydroxylation activity; ②, tyrosinase oxidation activity.

An integrative pathway of betalain biosynthesis was outlined according to differentially expressed transcripts (Figure 5). Two methods were used to collect genes involved in betalain biosynthesis pathway in *H. polyrhizus*. One is that the annotated transcripts were searched based on standard gene names and synonyms derived from comprehensive pathway from the KEGG pathway (http://www.kegg.jp/kegg-bin/search_pathway_text?) and a review from combined functional annotations. A total of 106 transcripts were assigned to the pathway (File S3A). The other way is that key gene sequences were downloaded from NCBI database to align with the two libraries by assembling *de novo*. Forty nine transcripts were aligned to the key genes of those published paper using *p* < 0.001 as a threshold (File S3B). Thirty-three transcripts related to betalain biosynthesis were identified from the white and red pulp transcriptome database (Table 2). Eighteen transcripts including tyrosine 3-monooxygenase, 4, 5-DOPA dioxygenase extradiol, cytochrome P450 and GT were significantly up-regulated expression. Fifteen were down-regulated including transcripts encoding TYR and DOPA decarboxylase.

**Table 2:** **Candidate transcripts related to betalains biosynthesis of Guanhuahong (*H. polyrhyzus*)**.

**Function**	**Gene**	**Enzyme**	**KO id (EC: No)**	**No. All^a^**	**No. Up^b^**	**No. Down^c^**
Betalains biosynthesis	*TYR*	tyrosinase	K00505 (1.14.18.1)	6	0	6
		tyrosine 3-monooxygenase	K06630 (1.10.3.1)	33	1	0
	*DOPA*	4,5-DOPA dioxygenase extradiol	K15777 (1.13.11.-)	13	8	1
	*6GT*	betanidin 6-O-glucosyltransferase	K15775 (2.4.1.-)	3	1	0
	*5GT*	betanidin 5-O-glucosyltransferase	K15774 (2.4.1.-)	2	0	0
	*CDOPA5GT*	cyclo-DOPA 5-O-glucosyltransferase	K15776 (2.4.1.-)	6	0	0
	*PaGT*	putative UDP-glucuronosyltransferase	K12930 (EC: 2.4.1.115)	3	1	0
	*CYP76AD*	cytochrome P450		20	6	0
	*COMT*	catechol O-methyltransferase	K00545 (2.1.1.6)	3	1	0
	*DDC*	aromatic-L-amino-acid decarboxylase/DOPA decarboxylase	K01593 (4.1.1.28)	17	0	8
	Total number of transcripts			106	18	15

a The total number of analyzed transcripts.

b The number of transcripts with significantly up-regulated expression in red pulp stage of H. polyrhizus compared with in white pulp stage.

c The number of transcripts with significantly down-regulated expression in red pulp stage of H. polyrhizus compared with in white pulp stage.

**Figure 6:**
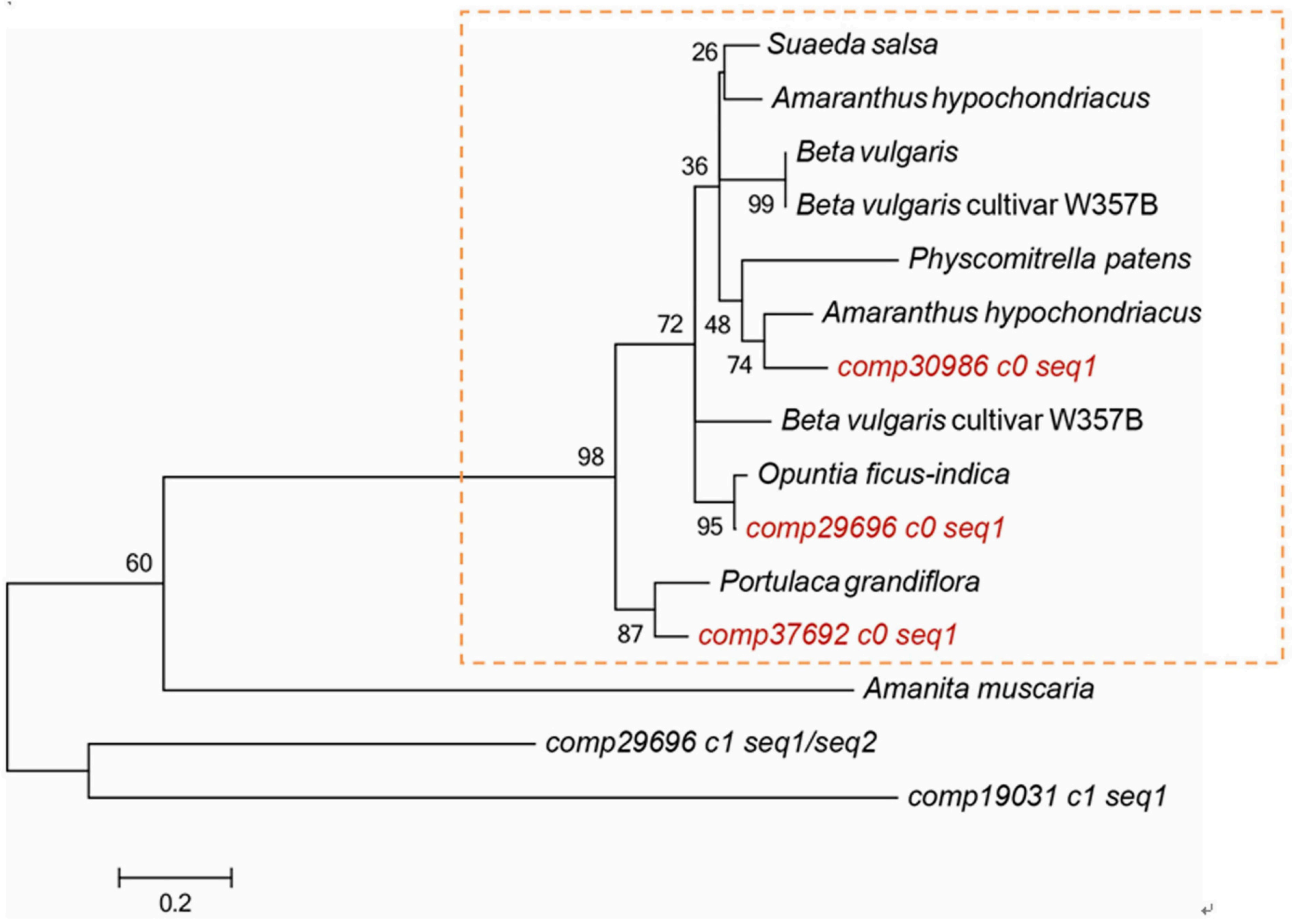
**Phylogenetic analysis of plant 4,5-DOPA-dioxygenases**. *Suaeda salsa*, ACO59903.1; *Amaranthus hypochondriacus*, ADZ48644.1; *Beta vulgaris*, CAE47100.1; *Beta vulgaris* cultivar W357B, AET43288.1/AET43293.1; *Physcomitrella patens*, CAE47099.1; *Amaranthus hypochondriacus*, AHV78224.1; *Opuntia ficus-indica*, ABW79880.1; *Portulaca grandiflora*, CAE45178.1.

Nine transcripts (*comp19031_c1_seq1, comp29696_c0_seq1, comp29696_c1_seq1, comp29696_c1_seq2, comp29696_c1_seq3, comp29696_c1_seq4, comp29696_c2_seq1, comp30986_c0_seq1, comp37692_c0_seq1*) annotated as DOD were obtained from the two libraries. They all were up-regulated expression in red pulp stage except *comp19031_c1_seq1* (File S3C). Phylogenetic analysis showed that *comp37692_c0_seq1, comp29696_c0_seq1*, and *comp30986_c0_seq1* belonged to the same type related to betalain biosynthesis. By contrast, *comp29696_c1_seq1, comp29696_c1_seq2*, and *comp19031_c1_seq1* shared relatively low similarity with the other transcripts (Figure 6). These results suggested that *comp37692_c0_seq1, comp29696_c0_seq1*, and *comp30986_c0_seq1* are putative candidate transcripts encoding DOD.

The roles of TYR were divided into two branches in betalain biosynthetic pathway i.e., dopaxanthin and cyclo-DOPA syntheses. Seven transcripts were identified from the transcriptome database. Among the 7 transcripts, 6 down-regulated transcripts (*comp37375_c0_seq1, comp37375_c0_seq2, comp37375_c0_seq3, comp37375_c0_seq4, comp37375_c0_seq5, comp37375_c0_seq6*) were hypothesized to be polyphenol oxidase (equivalent TYR) while one up-regulated transcript (*comp37674_c0_seq1*) was annotated as tyrosine 3-monooxygenase (equivalent tyrosine hydroxylase) (File S3C).

Six transcripts (*comp16058_c0_seq1, comp35191_c2_seq3, comp35191_c2_seq1, comp35191_c2_seq2, comp36993_c0_seq3, comp36993_c0_seq4*) were up-regulated expression from white pulp to red pulp stage (File S3C). *Comp16058_c0_seq1* had 239-fold changes and showed 74% identity to *Amaranthus cruentus CYP76AD2* (AET43291.1). *Comp36993_c0_seq3* and *comp36993_c0_seq4* showed 51 and 49% to *CYP76AD3* (AET43292.1) of *Mirabilis jalapa*, respectively. The rest three transcripts (*comp35191_c2_seq1, comp35191_c2_seq2*, and *comp35191_c2_seq3*) are similar to each other and had about 50% identity to *CYP76AD2* (AET43291.1) of *Amaranthus cruentus*.

Twelve transcripts were obtained based on reported results of *B5GT, B6GT*, and *CDOPA5GT*. Only one transcripts (*comp32889_c0_seq1*) encoding *B6GT* had a relatively higher expression level in red than white stage (File S3A). Expression levels of *B5GT* (2 transcripts) and *CDOPA5GT* (6 transcripts) were relatively low from white to red pulp stage (File S3A). However, no significant difference in *B5GT* and *CDOPA5GT* was detected between the two stages.

Eight transcripts encoding *DDC* in decarboxylated betalains biosynthesis had down-regulated expression patterns. Very low expression levels of *comp38514_c0_seq1* and *comp38987_c0_seq1* were detected in the two stages, suggesting they are irrelevant to betalain biosynthesis. COMT was encoded by up-regulated transcripts (*comp32369_c0_seq2*). But *comp32369_c0_seq2* had a low expression level in the two stages (File S3C).

As a whole, a preliminary pathway including some candidate transcripts was proposed according to betalain analyses and differentially expressed transcripts in combination with published documents (Figure 1, File S4, and Figure 5). Most up-regulated transcripts were mainly focused in one pathway and distributed on DOD, cytochrome P450 and GT (Figure 5).

### Verification of the accuracy of the RNA-Seq data using RT-qPCR

**Figure 7:**
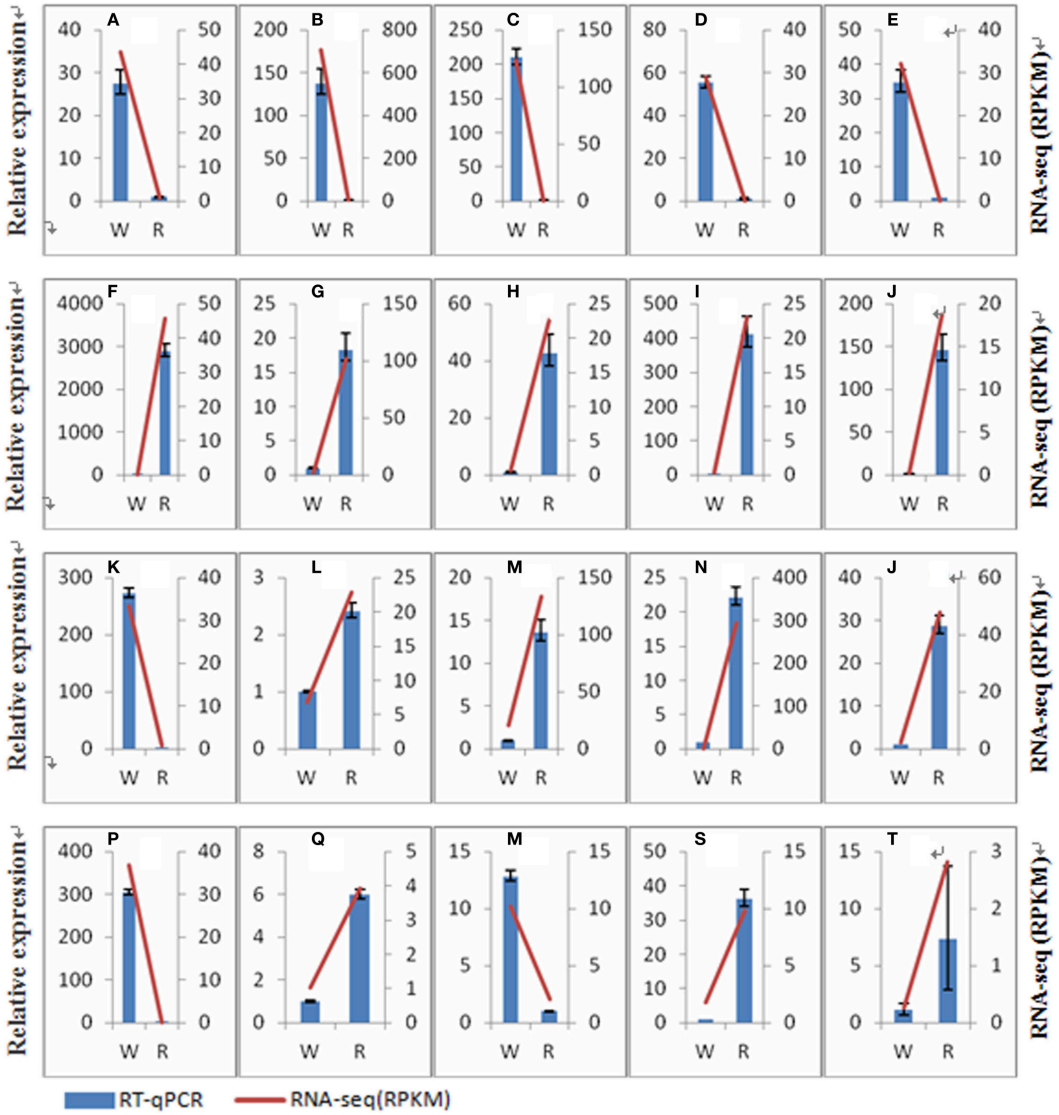
**Comparison of transcripts expression results from RNA-seq and RT-qPCR analyses**. W, white pulp stage of Guanhuahong; R, red pulp stage of Guanhuahong. **(A)**, *comp26707_c0_seq1*; **(B)**, *comp37672_c0_seq1*; **(C)**, *comp37717_c0_seq1*; **(D)**, *comp28147_c0_seq1*; **(E*)****, comp23614_c0_seq1*; **(F)**, *comp29487_c0_seq1*; **(G)**, *comp33513_c0_seq1*; **(H)**, *comp32700_c0_seq1*; **(I)**, *comp34286_c2_seq1*; **(J)**, comp30867_c0_seq1; **(K)**, *comp37375_c0_seq2*; **(L)**, comp30986_c0_seq1; **(M)**, *comp37692_c0_seq1*; **(N)**, *comp16058_c0_se*q1; **(O)**, *comp36993_c0_seq4*; **(P)**, *comp24898_c0_seq1*; **(Q)**, *comp32369_c0_seq2*; **(R)**, *comp37261_c1_seq14*; **(S)**, *comp36238_c0_seq2*; **(T)**, *comp35191_c2_seq2*.

Ten unknown transcripts with significant difference between the two stages and 14 transcripts involved in betalain biosynthesis were selected to validate the accuracy of RNA-Seq data by RT-qPCRs (Figure 7). Expression patterns of these 24 transcripts were consistent with the RNA-Seq data. These results suggested that RNA-Seq data is credible and can be used for subsequent experiments. The IDs, RPKM value (Reads per kilobase of exon model per million mapped read), primers of the 24 transcripts were shown in File S3 and Table S1.

### Expression of candidate transcripts related to betalain biosynthesis at all pulp coloration stages

**Figure 8:**
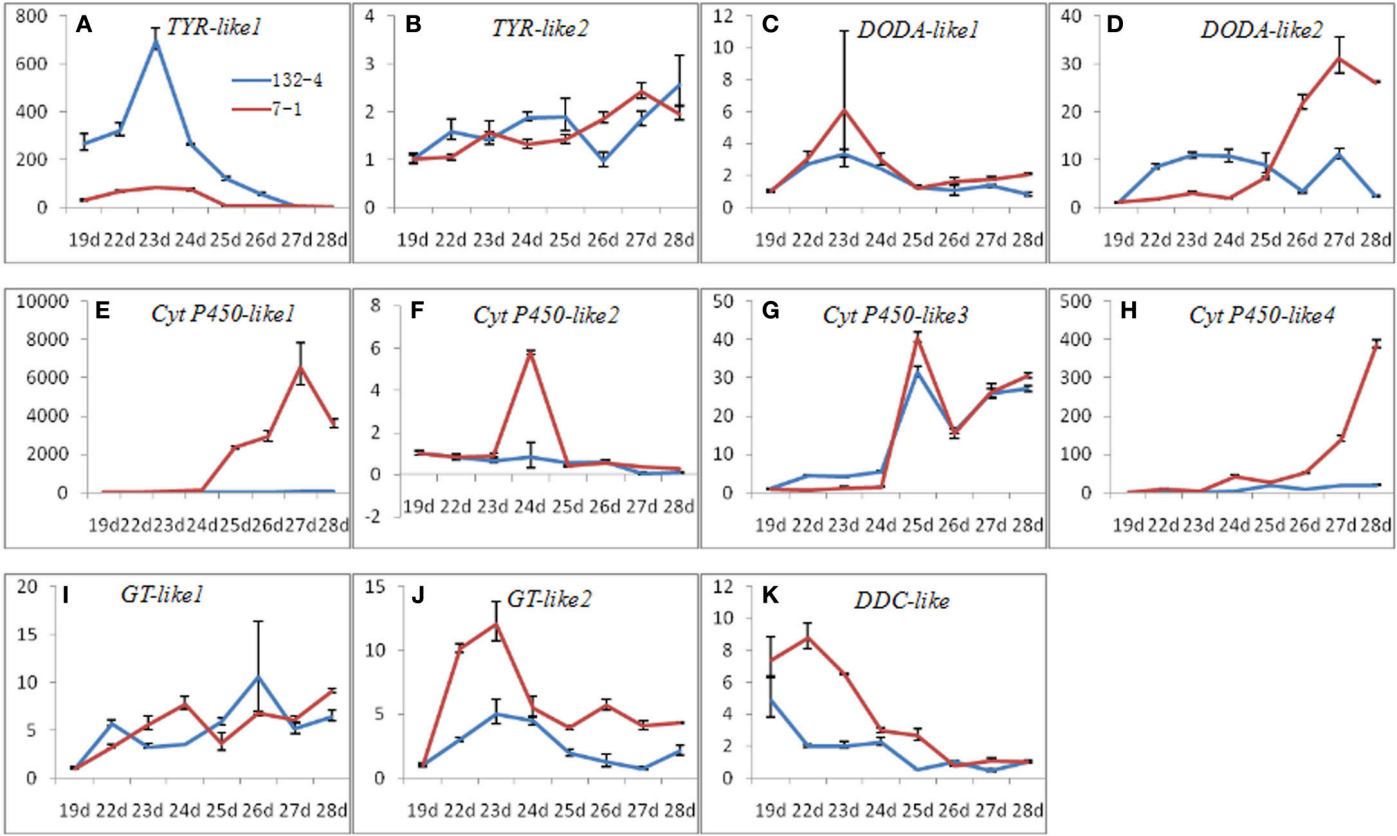
**Expression analyses of candidate transcripts related to betalain biosynthesis at all pulp coloration stages of 132-4 (*H. undatus*) and 7-1 (*H. polyrhizus*) using RT-qPCR**. **(A)**, *comp37375_c0_seq2*; **(B)**, *comp37674_c0_seq1*; **(C)**, *comp30986_c0_seq1*; **(D)**, *comp37692_c0_seq1*; **(E)**, *comp16058_c0_seq1*; **(F)**, *comp35191_c2_seq2*; **(G)**, *comp36993_c0_seq3*; **(H)**, *comp36993_c0_seq4*; **(I)**, *comp36238_c0_seq2*; **(J)**, *comp32889_c0_seq1*; **(K)**, *comp37261_c1_seq14*.

To elucidate roles of candidate transcripts in betalain biosynthetic pathway, the expression levels of candidate transcripts related to betalain biosynthesis were detected at all stages of pulp coloration in *H. polyrhizus* and *H. undatus*. Most transcripts had higher expression levels in red pitaya except *comp37375_c0_seq2* (Figure 8). Higher expression level of *comp37375_c0_seq2* was observed in *H. undatus* than *H. polyrhizus* (Figure 8A). Three expression patterns i.e., increasing, increasing at first and decreasing thereafter, and decreasing were detected at all pulp coloration stages of *H. polyrhizus* and *H. undatus*.

Cyt P450 (*comp36993_c0_seq4*) was up-regulated during pulp coloration in *H. polyrhizus* compared to stable and lower in *H. undatus* (Figure 8H). TYR (*comp37375_c0_seq2*), DOD (*comp37692_c0_seq1* and *comp30986_c0_seq1*), B6GT (*comp32889_c0_seq1*) and cyt P450 (*comp16058_c0_seq1* and *comp35191_c2_seq2*) had the same expression trends (increasing at first and decreasing thereafter) at all pulp coloration stages of *H. polyrhizus* (Figures 8A–F,J). The highest expression levels of *comp37375_c0_seq2, comp35191_c2_seq2, comp32889_c0_seq1*, and *comp30986_c0_seq1* were detected at the early stage. The maximum expression levels of *comp37375_c0_seq2, comp32889_c0_seq1*, and *comp30986_c0_seq1* were detected in *H. polyrhizus* and *H. undatus* on the 23^*rd*^ DAAP (pulp color begins to change from white to red) compared to *comp35191_c2_seq2* on the 24^th^ DAAP (Figures 8A,C,F,J). By contrast, *comp37692_c0_seq1* and *comp16058_c0_seq1* were up-regulated expression at the early stages of pulp coloration and declined slightly at the late stages of pulp coloration (Figures 8D,E). DDC (*comp37261_c1_seq14*) showed a decreasing trend at all pulp coloration stages and higher expression levels were detected in *H. polyrhizus* than *H. undatus* (Figure 8K). *Comp37674_c0_seq1, comp36238_c0_seq2*, and *comp36993_c0_seq3* had up-regulated trends at all pulp coloration stages in *H. polyrhizus* and *H. undatus*. However, no significant difference was detected between *H. polyrhizus* and *H. undatus* (Figures 8B,I,G).

## Discussion

Betalains have attracted much attention of the world due to their high nutritional value and bioactivities. However, except for the cloning of single gene in the betalain pathway in model plant, the biosynthetic pathway of betalains remains to be fully clarified. In the present study, transcriptome sequencing was performed to screen genes related to betalain biosynthesis from *H. polyrhizus*. A preliminary pathway (Figure 5) was proposed based on betalain analyses and expression analyses of candidate transcripts in combination with published documents. Genes involved in each step of the betalain biosynthetic pathway were obtained from the transcriptome dataset, suggesting that it had high coverage. Higher expression levels of transcripts related to betalains were detected in *H. polyrhizus* and 9 transcripts were involved in betalain biosynthesis.

TYR played a primary role in the initial betalain biosynthetic steps. Positive correlations between betacyanin contents and TYR activities were found in *P. grandiflora, B. vulgaris*, and *S. salsa* (Steiner et al., 1999; Gandia-Herrero et al., 2004; Wang et al., 2007). In this study, two transcripts (*comp37674_c0_seq1* and *comp37375_c0_seq2*) encoding tyrosine hydroxylase were obtained from the two libraries. Different expression patterns of *comp37674_c0_seq1* and *comp37375_c0_seq2* were observed. *Comp37674_c0_seq1* showed up-regulated trends at all pulp coloration stages in *H. polyrhizus* and *H. undatus* (Figure 8B) suggesting TYR hydroxylation activity played equivalent function in *H. polyrhizus* and *H. undatus*. Similar results were obtained in *H. polyrhizus* and *H. undatus* according to metabolite profiling of L-DOPA (Suh et al., 2014). *Comp37375_c0_seq2* increased gradually before 23 DAAP and reduced thereafter in *H. polyrhizus* and *H. undatus* (Figure 8A). The results were consistent with founding that TYR hydroxylation activity was increased at first and then decreased, with rising of betacyanin contents during seedling development of *B. vulgaris* (Steiner et al., 1999). TYR-formed dopa must be protected from the oxidase activity of TYR so that it could be used as substrate of DOD (Steiner et al., 1999). In our study, higher expression level of *comp37375_c0_seq2* was detected in *H. undatus* than *H. polyrhizus* (Figure 8A) indicating that *comp37375_c0_seq2* keeps high oxidation activity in *H. undatus* to reduce L-DOPA activity.

*Cytochrome P450* gene encodes a key enzyme in the betalain synthesis pathway (Hatlestad et al., 2012; Yang et al., 2015). It can convert L-DOPA into cyclo-DOPA which is required for red betalain production in *B. Vulgaris* (Hatlestad et al., 2012). In our study, *three* transcripts (*comp16058_c0_seq1, comp36993_c0_seq4*, and *comp35191_c2_seq2*) encoding cyt-P450 was achieved from the two libraries. The expression level of *comp16058_c0_seq1* increased gradually during pulp color transition from white to red stages and decreased at full maturation stage. Higher expression levels of *comp16058_c0_seq1* were detected in *H. polyrhizus* than *H. undatus* (Figure 8E). Similar expression pattern of *comp36993_c0_seq4* was also observed in *H. polyrhizus* during the whole pulp coloration stages, but its expression level was lower than that of *comp16058_c0_seq1* (Figure 8H). It was reported that expression level of *CYP76ADs* was related to betalains in *B. vulgaris* (Hatlestad et al., 2012), *Amaranthus hypochondriacus* (Casique-Arroyo et al., 2014) and *Mirabilis jalapa* (Suzuki et al., 2014). In this study, betacyanin, and betaxanthin contents were increasing at all stages of pulp coloration in *H. polyrhizus* (Figure 1). Expression levels of *comp16058_c0_seq1* and *comp36993_c0_seq4* were positive correlation with betalain contents (Figure 1). In addition, *comp35191_c2_seq2* had undergone a sharp increase and a dramatic decrease from 23^rd^ to 25^th^ day (Figure 8F), indicating that *comp35191_c2_seq2* is an inducible gene involved in betalain pathway and contributed to the early stage. Those results suggested that *comp16058_c0_seq1* is an important candidate gene of cyt-P450 involved in betalain biosynthesis. Further work is being carried out to understand its roles in betalain biosynthesis of *H. polyrhizus* via genetic transformation.

*DOD* is a key enzyme contributed to 4, 5-seco-DOPA (the precursor of betalamic acid). The expression level of *DOD* was positive correlation with betalains in *B. vulgaris* (Gandia-Herrero and Garcia-Carmona, 2012) and *Parakeelya* (Chung et al., 2015). In the present study, similar trend was observed between expression level of *DOD* and betalain accumulations (Figures 1, 8D). Lower expression levels of *DOD* (*comp37692_c0_seq1* and *comp30986_c0_seq1*) were detected in *H. undatus* while higher expression levels of *comp37692_c0_seq1* were observed at late stage of pulp coloration in *H. polyrhizus* compared to lower at early stage (Figures 8C,D). The expression of *comp37692_c0_seq1* is higher than *comp30986_c0_seq1* nearly in all stages. The expression trends of *comp37692_c0_seq1* and *comp30986_c0_seq1* are consistent with *comp16058_c0_seq1* (cyt-P450) and *comp35191_c2_seq2* (cyt-P450), respectively. Therefore, *comp37692_c0_seq1* and *comp30986_c0_seq1* are considered as candidate genes of *DOD*.

GTs catalyze the transferring of sugar moieties to form more complex betacyanins and keep stability of generated pigments (Gandia-Herrero and Garcia-Carmona, 2013). Previous studies showed there are various betacyanins in red pitaya and several glycosylation reactions were predicted (Suh et al., 2014). In this study, 14 transcripts annotated GT were obtained from transcriptome data and only two transcripts (c*omp32889_c0_seq1* and *comp36238_c0_seq2*) were up-regulated in the red stage (File S3C). The result is not consistent with the above-mentioned conjecture. Furthermore, the expression trends of the two transcripts were not accordant with the betalain contents during the eight stages (Figures 1, 8I,G). The reason may be some GTs contributed to be betacyanins have not been identified.

Aromatic-L-amino-acid decarboxylase (DDC, equivalent Tyrosine/DOPA decarboxylase) and catechol O-methyltransferase (COMT) are involved in the formation of decarboxylated betalains (Gandia-Herrero and Garcia-Carmona, 2013). In this study, DDC (*comp37261_c1_seq14*) showed a downtrend in white and red pitayas (Figure 8K). Negative correlation was detected between betalain contents and expression level of *comp37261_c1_seq14* (Figures 1, 8K) suggesting *comp37261_c1_seq14* isn't a key gene in betalain biosynthesis of pitaya. The result is also consistent with the report of low decarboxylated betalain contents in red pitaya (Suh et al., 2014).

## Conclusions

To date, pitaya is increasingly gaining the public attention due to its high nutritional value and strong antioxidant properties. This study presented the first transcriptome of *H. polyrhizus*. The large RNA-Seq data could provide valuable information concerning betalain biosynthesis in red pitaya. A total of about 12 Gb raw RNA-Seq data was generated and *de novo* assembled into 122,677 transcripts, in which 122,668 were annotated. The putative betalain-related genes were identified and characterized in the pulp coloration process of pitaya. Based on betalain analyses, expression analyses of candidate transcripts and published documents, we proposed a preliminary pathway of betalain biosynthesis in pitaya. Further work such as enzyme activity analyses and genetic transformation are being carried out to elucidate their roles in betalain biosynthesis of pitaya.

## Author contributions

Conceived and designed the experiments: HQ and QY. Performed the experiments: HQ. Analyzed the data: CC, CZ, HG, and QY. Contributed reagents/materials/analysis tools: CP, MY, WJ, ZJ, HG, and ZJ. Wrote the paper: HQ and QY.

### Conflict of interest statement

The authors declare that the research was conducted in the absence of any commercial or financial relationships that could be construed as a potential conflict of interest.

## Supplementary material

Figure S1 **Pitaya used in this study. (A)** White pulp of Guanhuahong (*H. polyrhyzus*) used for RNA-Seq. **(B)** Red pulp of Guanhuahong (*H. polyrhyzus*) used for RNA-Seq. **(C)** Changes in pulp color of 7-1 (*H. polyrhyzus*) at all fruit maturation stages. **(D)** Changes in pulp color of 132-4 (*H. undatus*) at all fruit maturation stages. Bars in **(A,B)** = 2 cm, **(C,D)** = 5 cm.

Figure S2 **Statistics of assembly length for transcripts**.

Figure S3 **GO and COG classification of transcripts. (A)**, GO categories of transcripts; **(B)**, COG classification of transcripts.

Table S1 **Primers used in this study**.

Table S2 **Expression statistics of transcripts between the white and red pulp libraries**.

File S1 **All transcripts between the white and red pulp libraries assigned to 309 KEGG pathways of *H. polyrhizus***.

File S2 **Significantly differentially expressed transcripts between the white and red pulp libraries assigned to 241 KEGG pathways of *H. polyrhizus***.

File S3 **Functional annotation and analyses of transcripts. (A)** Transcripts were searched based on standard gene names and synonyms. **(B)** Transcripts were achieved by aligning pitaya libraries with the key gene sequences downloaded from NCBI database. **(C)** All differentially expressed transcripts involved in betalain biosynthesis transcripts.

File S4 **Annotation and identification of some candidate transcript sequences from NCBI**.

## References

[B1] Adnan L. Osman A. Hamid A. A. (2011). Antioxidant activity of different extracts of red pitaya (*Hylocereus polyrhizus*) seed. Int. J. Food Prop. 14, 1171–1181. 10.1080/10942911003592787

[B2] Allegra M. Ianaro A. Tersigni M. Panza E. Tesoriere L. Livrea M. A. (2014). Indicaxanthin from cactus pear fruit exerts anti-inflammatory effects in carrageenin-induced rat pleurisy. J. Nutr. 144, 185–192. 10.3945/jn.113.183657 24306215

[B3] Altschul S. F. Gish W. (1996). Local alignment statistics. Method Enzymol. 266, 460–480. 10.1016/S0076-6879(96)66029-7 8743700

[B4] Altschul S. F. Madden T. L. Schaeffer A. A. Zhang J. Zhang Z. Miller W.. (1997). Gapped BLAST and PSI-BLAST: a new generation of protein database search programs. Nuleic Acids Res. 25, 3389–3402. 10.1093/nar/25.17.3389 9254694 PMC146917

[B5] Casique-Arroyo G. Martinez-Gallardo N. Gonzalez de la Vara L. Delano-Frier J. P. (2014). Betacyanin biosynthetic genes and enzymes are differentially induced by (a) biotic stress in *Amaranthus hypochondriacus*. PLoS ONE 9:e990126. 10.1371/journal.pone.0099012 24896616 PMC4045864

[B6] Christinet L. Burdet F. Zaiko M. Hinz U. Zryd J. P. (2004). Characterization and functional identification of a novel plant 4,5-extradiol dioxygenase involved in betalain pigment biosynthesis in *Portulaca grandiflora*. Plant Physiol. 134, 265–274. 10.1104/pp.103.031914 14730069 PMC316306

[B7] Chung H. Schwinn K. E. Ngo H. M. Lewis D. H. Massey B. Calcottt K. E.. (2015). Characterisation of betalain biosynthesis in *Parakeelya* flowers identifies the key biosynthetic gene DOD as belonging to an expanded LigB gene family that is conserved in betalain-producing species. Front Plant Sci. 6:499. 10.3389/fpls.2015.00499 26217353 PMC4493658

[B8] Clifford T. Howatson G. West D. J. Stevenson E. J. (2015). The potential benefits of red beetroot supplementation in health and disease. Nutrients 7, 2801–2822. 10.3390/nu7042801 25875121 PMC4425174

[B9] Esquivel P. Stintzing F. C. Carle R. (2007). Pigment pattern and expression of colour in fruits from different *Hylocereus sp* genotypes. Innov. Food Sci. Emerg. 8, 451–457. 10.1016/j.ifset.2007.03.022

[B10] Gandia-Herrero F. Escribano J. Garcia-Carmona F. (2005b). Betaxanthins as substrates for tyrosinase. An approach to the role of tyrosinase in the biosynthetic pathway of betalains. Plant Physiol. 138, 421–432. 10.1104/pp.104.057992 15805475 PMC1104195

[B11] Gandia-Herrero F. Escribano J. García-Carmona F. (2005a). Characterization of the monophenolase activity of tyrosinase on betaxanthins: the tyramine-betaxanthin/dopamine-betaxanthin pair. Planta 222, 307–318. 10.1007/s00425-005-1526-4 15968512

[B12] Gandia-Herrero F. Garcia-Carmona F. (2012). Characterization of recombinant *Beta vulgaris* 4, 5-DOPA-extradiol- dioxygenase active in the biosynthesis of betalains. Planta 236, 91–100. 10.1007/s00425-012-1593-2 22270561

[B13] Gandia-Herrero F. Garcia-Carmona F. (2013). Biosynthesis of betalains: yellow and violet plant pigments. Trends Plant Sci. 18, 334–343. 10.1016/j.tplants.2013.01.003 23395307

[B14] Gandia-Herrero F. Garcia-Carmona F. Escribano J. (2004). Purification and characterization of a latent polyphenol oxidase from beet root (*Beta vulgaris* L.). J. Agr. Food Chem. 52, 609–615. 10.1021/jf034381m 14759157

[B15] Garcia-Cruz L. Valle-Guadarrama S. Salinas-Moreno Y. Joaquin-Cruz E. (2013). Physical, chemical, and antioxidant activity characterization of pitaya (*Stenocereus pruinosus*) fruits. Plant Food Hum. Nutr. 68, 403–410. 10.1007/s11130-013-0391-8 24142131

[B16] Grabherr M. G. Haas B. J. Yassour M. Levin J. Z. Thompson D. A. Amit I.. (2011). Full-length transcriptome assembly from RNA-Seq data without a reference genome. Nat. Biotechnol. 29, 644–652. 10.1038/nbt.1883 21572440 PMC3571712

[B17] Harris M. A. Clark J. Ireland A. Lomax J. Ashburner M. Foulger R.. (2004). Gene Ontology Consortium. The Gene Ontology (GO) database and informatics resource. Nucleic Acids Res. 32, D258–D261. 10.1093/nar/gkh036 14681407 PMC308770

[B18] Hatlestad G. J. Akhavan N. A. Sunnadeniya R. M. Elam L. Cargile S. Hembd A.. (2015). The beet Y locus encodes an anthocyanin MYB-like protein that activates the betalain red pigment pathway. Nat. Genet. 47, 92–96. 10.1038/ng.3163 25436858

[B19] Hatlestad G. J. Sunnadeniya R. M. Akhavan N. A. Gonzalez A. Goldman I. L. McGrath J. M.. (2012). The beet R locus encodes a new cytochrome P450 required for red betalain production. Nat. Genet. 44, 816–820. 10.1038/ng.2297 22660548

[B20] Hind G. Marshak D. R. Coughlan S. J. (1995). Spinach thylakoid polyphenol oxidase: cloning, characterization, and relation to a putative protein kinase. Biochemistry 34, 8157–8164. 10.1021/bi00025a022 7794929

[B21] Isayenkova J. Wray V. Nimtz M. Strack D. Vogt T. (2006). Cloning and functional characterisation of two regioselective flavonoid glucosyltransferases from *Beta vulgaris*. Phytochemistry 67, 1598–1612. 10.1016/j.phytochem.2006.06.026 16876834

[B22] Joy R. W. I. Sugiyama M. Fukuda H. Komamine A. (1995). Cloning and characterization of polyphenol oxidase cDNAs of *Phytolacca americana*. Plant Physiol. 107, 1083–1089. 10.1104/pp.107.4.1083 7539531 PMC157240

[B23] Kanehisa M. Goto S. Hattori M. Aoki-Kinoshita K. F. Itoh M. Kawashima S.. (2006). From genomics to chemical genomics: new developments in KEGG. Nucleic Acids Res. 34, D354–D357. 10.1093/nar/gkj102 16381885 PMC1347464

[B24] Lee E. J. An D. Nguyen C. T. T. Patil B. S. Kim J. Yoo K. S. (2014). Betalain and betaine composition of greenhouse- or field-produced beetroot (*Beta vulgaris* L.) and inhibition of HepG2 cell proliferation. J. Agr. Food Chem. 62, 1324–1331. 10.1021/jf404648u 24467616

[B25] Lim S. D. Yusof Y. A. Chin N. L. Talib R. A. Endan J. Aziz M. G. (2011). Effect of extraction parameters on the yield of betacyanins from pitaya fruit (*Hylocereus polyrhizus*) pulps. J. Food Agr. Environ. 9, 158–162.

[B26] Ma H. Zhu H. Y. Li L. L. Chen L. J. Guo Z. F. Zhong M. (2013). Cloning and sequence analysis of polyphenol oxidase gene of *Suaeda salsa*. Guangdong Agr. Sci. 40, 149–152. 10.3969/j.issn.1004-874X.2013.11.041

[B27] Martinez R. M. Longhi-Balbinot D. T. Zarpelon A. C. Staurengo-Ferrari L. Baracat M. M. Georgetti S. R.. (2015). Anti-inflammatory activity of betalain-rich dye of *Beta vulgaris*: effect on edema, leukocyte recruitment, superoxide anion and cytokine production. Arch Pharm. Res. 38, 494–504. 10.1007/s12272-014-0473-7 25173360

[B28] Naderi N. Stintzing F. C. Ghazali H. M. Manap Y. A. Jazayeri S. D. (2010). Betalain extraction from *Hylocereus polyrhizus* for natural food coloring purposes. J. Prof. Assoc. Cactus. 12, 143–154.

[B29] Nakatsuka T. Yamada E. Takahashi H. Imamura T. Suzuki M. Ozeki Y.. (2013). Genetic engineering of yellow betalain pigments beyond the species barrier. Sci. Rep. 3:1970. 10.1038/srep01970 23760173 PMC3679504

[B30] Noguchi A. Kunikane S. Homma H. Liu W. Sekiya T. Hosoya M. (2009). Identification of an inducible glucosyltransferase from *Phytolacca americana* L. cells that are capable of glucosylating capsaicin. Plant Biotechnol. 26, 285–292. 10.5511/plantbiotechnology.26.285

[B31] Rebecca O. P. S. Boyce A. N. Chandran S. (2010a). Pigment identification and antioxidant properties of red dragon fruit (*Hylocereus polyrhizus*). Afr. J. Biotechnol. 9, 1450–1454.

[B32] Rebecca O. P. S. Harivaindaran K. V. Boyce A. N. Chandran S. (2010b). Potential natural dye with antioxidant properties from red dragon fruit (*Hylocereus polyrhizus*). Acta Horticul. 875, 477–485. 10.17660/ActaHortic.2010.875.62

[B33] Robinson M. D. McCarthy D. J. Smyth G. K. (2010). edgeR: a Bioconductor package for differential expression analysis of digital gene expression data. Bioinformatics. 26, 139–140. 10.1093/bioinformatics/btp616 19910308 PMC2796818

[B34] Ruan Y. (2008). The Cloning and Functional Analysis of 4, 5-DOPA-dioxygenase in Suaeda salsa. Jinan: Shandong Normal University.

[B35] Sasaki N. Abe Y. Goda Y. Adachi T. Kasahara K. Ozeki Y. (2009). Detection of DOPA 4,5-Dioxygenase (DOD) activity using recombinant protein prepared from *Escherichia coli* cells harboring cDNA encoding DOD from *Mirabilis jalapa*. Plant Cell Physiol. 50, 1012–1016. 10.1093/pcp/pcp053 19366710

[B36] Sasaki N. Wada K. Koda T. Kasahara K. Adachi T. Ozeki Y. (2005). Isolation and characterization of cDNAs encoding an enzyme with glucosyltransferase activity for cyclo-DOPA from four o'clocks and feather cockscombs. Plant Cell Physiol. 46, 666–670. 10.1093/pcp/pci064 15695438

[B37] Sepulveda-Jimenez G. Rueda-Benitez P. Porta H. Rocha-Sosa M. (2004). Betacyanin synthesis in red beet (*Beta vulgaris*) leaves induced by wounding and bacterial infiltration is preceded by an oxidative burst. Physiol Mol. Plant P. 64, 125–133. 10.1016/j.pmpp.2004.08.003

[B38] Shimada S. Inoue Y. T. Sakuta M. (2005). Anthocyanidin synthase in non-anthocyanin-producing caryophyllales species. Plant J. 44, 950–959. 10.1111/j.1365-313X.2005.02574.x 16359388

[B39] Stafford H. A. (1994). Anthocyanins and betalains: evolution of the mutually exclusive pathways. Plant Sci. 101, 91–98. 10.1016/0168-9452(94)90244-5

[B40] Steiner U. Schliemann W. Boehm H. Strack D. (1999). Tyrosinase involved in betalain biosynthesis of higher plants. Planta 208, 114–124. 10.1007/s004250050541

[B41] Stintzing F. C. Herbach K. M. Mosshammer M. R. Carle R. Yi W. G. Sellappan S.. (2005). Color, betalain pattern, and antioxidant properties of cactus pear (*Opuntia* spp.) clones. J. Agr. Food Chem. 53, 442–451. 10.1021/jf048751y 15656686

[B42] Stintzing F. C. Schieber A. Carle R. (2002). Identification of betalains from yellow beet (*Beta vulgaris* L.) and cactus pear [*Opuntia ficus-indica* (L.) Mill.] by high-performance liquid chromatography-electrospray ionization mass spectrometry. J. Agr. Food Chem. 50, 2302–2307. 10.1021/jf011305f 11929288

[B43] Strack D. Vogt T. Schliemann W. (2003). Recent advances in betalain research. Phytochemistry 62, 247–269. 10.1016/S0031-9422(02)00564-2 12620337

[B44] Stracke R. Holtgrawe D. Schneider J. Pucker B. Sorensen T. R. Weisshaar B. (2014). Genome-wide identification and characterisation of R2R3-MYB genes in sugar beet (*Beta vulgaris*). BMC Plant Biol. 14:249. 10.1186/s12870-014-0249-8 25249410 PMC4180131

[B45] Suh D. H. Lee S. Heo D. Y. Kim Y. Cho S. K. Lee S.. (2014). Metabolite profiling of red and white pitayas (*Hylocereus polyrhizus* and *Hylocereus undatus*) for comparing betalain biosynthesis and antioxidant activity. J. Agr. Food Chem. 62, 8764–8771. 10.1021/jf5020704 25101804

[B46] Suzuki M. Miyahara T. Tokumoto H. Hakamatsuka T. Goda Y. Ozeki Y.. (2014). Transposon-mediated mutation of CYP76AD3 affects betalain synthesis and produces variegated flowers in four o'clock (*Mirabilis jalapa*). J. Plant Physiol. 171, 1586–1590. 10.1016/j.jplph.2014.07.010 25151127

[B47] Swarna J. Lokeswari T. S. Smita M. Ravindhran R. (2013). Characterisation and determination of *in vitro* antioxidant potential of betalains from *Talinum triangulare* (Jacq.) Willd. Food Chem. 141, 4382–4390. 10.1016/j.foodchem.2013.06.108 23993629

[B48] Takahashi K. Takamura E. Sakuta M. (2009). Isolation and expression analysis of two DOPA dioxygenases in *Phytolacca americana*. Z. Naturforsch. C 64, 564–573. 10.1515/znc-2009-7-816 19791510

[B49] Tanaka Y. Sasaki N. Ohmiya A. (2008). Biosynthesis of plant pigments: anthocyanins, betalains and carotenoids. Plant J. 54, 733–749. 10.1111/j.1365-313X.2008.03447.x 18476875

[B50] Tatusov R. L. Fedorova N. D. Jackson J. D. Jacobs A. R. Kiryutin B. Koonin E. V.. (2003). The COG database: an updated version includes eukaryotes. BMC Bioinformatics. 4:41. 10.1186/1471-2105-4-41 12969510 PMC222959

[B51] Tatusov R. L. Koonin E. V. Lipman D. J. (1997). A genomic perspective on protein families. Science 278, 631–637. 10.1126/science.278.5338.631 9381173

[B52] Tenore G. C. Novellino E. Basile A. (2012). Nutraceutical potential and antioxidant benefits of red pitaya (*Hylocereus polyrhizus*) extracts. J. Funct. Foods. 4, 129–136. 10.1016/j.jff.2011.09.003

[B53] Vogt T. (2002). Substrate specificity and sequence analysis define a polyphyletic origin of betanidin 5- and 6-O-glucosyltransferase from *Dorotheanthus bellidiformis*. Planta 214, 492–495. 10.1007/s00425-001-0685-1 11855654

[B54] Vogt T. Grimm R. Strack D. (1999). Cloning and expression of a cDNA encoding betanidin 5-O-glucosyltransferase, a betanidin- and flavonoid-specific enzyme with high homology to inducible glucosyltransferases from the Solanaceae. Plant J. 19, 509–519. 10.1046/j.1365-313X.1999.00540.x 10504573

[B55] Wang C. Q. Song H. Gong X. Z. Hu Q. G. Liu F. Wang B. S. (2007). Correlation of tyrosinase activity and betacyanin biosynthesis induced by dark in C-3 halophyte *Suaeda salsa* seedlings. Plant Sci. 173, 487–494. 10.1016/j.plantsci.2007.07.010

[B56] Woo K. K. Wong F. N. F. Chua H. S. C. Tang P. Y. (2011). Stability of the spray-dried pigment of red dragon fruit [*Hylocereus polyrhizus* (Weber) Britton and Rose] as a function of organic acid additives and storage conditions. Philipp Agr. Sci. 94, 264–269.

[B57] Wu L. C. Hsu H. W. Chen Y. C. Chiu C. C. Lin Y. I. Ho J. A. A. (2006). Antioxidant and antiproliferative activities of red pitaya. Food Chem. 95, 319–327. 10.1016/j.foodchem.2005.01.002

[B58] Wybraniec S. Stalica P. Jerz G. Klose B. Gebers N. Winterhalter P.. (2009). Separation of polar betalain pigments from cacti fruits of *Hylocereus polyrhizus* by ion-pair high-speed countercurrent chromatography. J. Chromatogr A. 1216, 6890–6899. 10.1016/j.chroma.2009.08.035 19732900

[B59] Yan F. X. Wen X. P. Gao G. L. Tao J. (2013). Cloning and sequence analysis of housekeeping genes *Actin* and *UBQ* from pitaya. Guizhou Agr. Sci. 41, 1–4. 10.3969/j.issn.1001-3601.2013.09.001

[B60] Yang Y. Moore M. J. Brockington S. F. Soltis D. E. Wong G. K. Carpenter E. J.. (2015). Dissecting molecular evolution in the highly diverse plant clade Caryophyllales using transcriptome sequencing. Mol. Biol. Evol. 32, 2001–2014. 10.1093/molbev/msv081 25837578 PMC4833068

[B61] Zdobnov E. M. Apweiler R. (2001). InterProScan. An integration platform for the signature-recognition methods in InterPro. Bioinformatics 17, 847–848. 10.1093/bioinformatics/17.9.847 11590104

[B62] Zhao S. Z. Sun H. Z. Gao Y. Sui N. Wang B. S. (2011). Growth regulator-induced betacyanin accumulation and dopa-4,5-dioxygenase (DODA) gene expression in euhalophyte *Suaeda salsa* calli. In Vitro Cell Dev. Pl. 47, 391–398. 10.1007/s11627-011-9339-6

