# Supplementary figures and images for: Transcriptomic Analysis Reveals Key Genes Related to Betalain Biosynthesis in Pulp Coloration of Hylocereus polyrhizus
Source: Front Plant Sci. 2016 Jan 5;6:1179. doi: 10.3389/fpls.2015.01179 (PMC4700300; doi:10.3389/fpls.2015.01179)

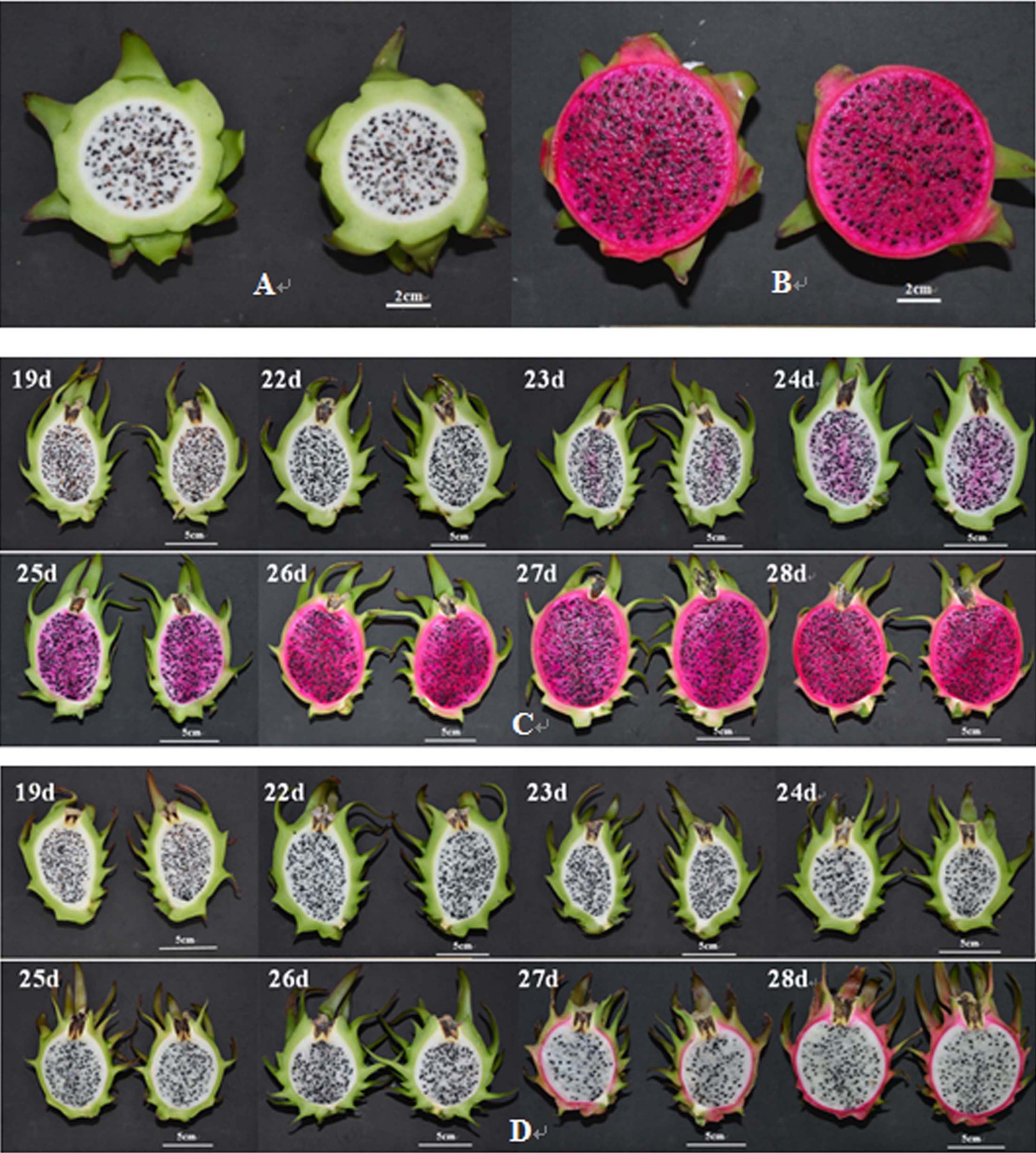

Supplement: Figure S1 — Pitaya used in this study. (A) White pulp of Guanhuahong (H. polyrhyzus) used for RNA-Seq. (B) Red pulp of Guanhuahong (H. polyrhyzus) used for RNA-Seq. (C) Changes in pulp color of 7-1 (H. polyrhyzus) at all fruit maturation stages. (D) Changes in pulp color of 132-4 (H. undatus) at all fruit maturation stages. Bars in (A,B) = 2 cm, (C,D) = 5 cm. [file Image1.JPEG]

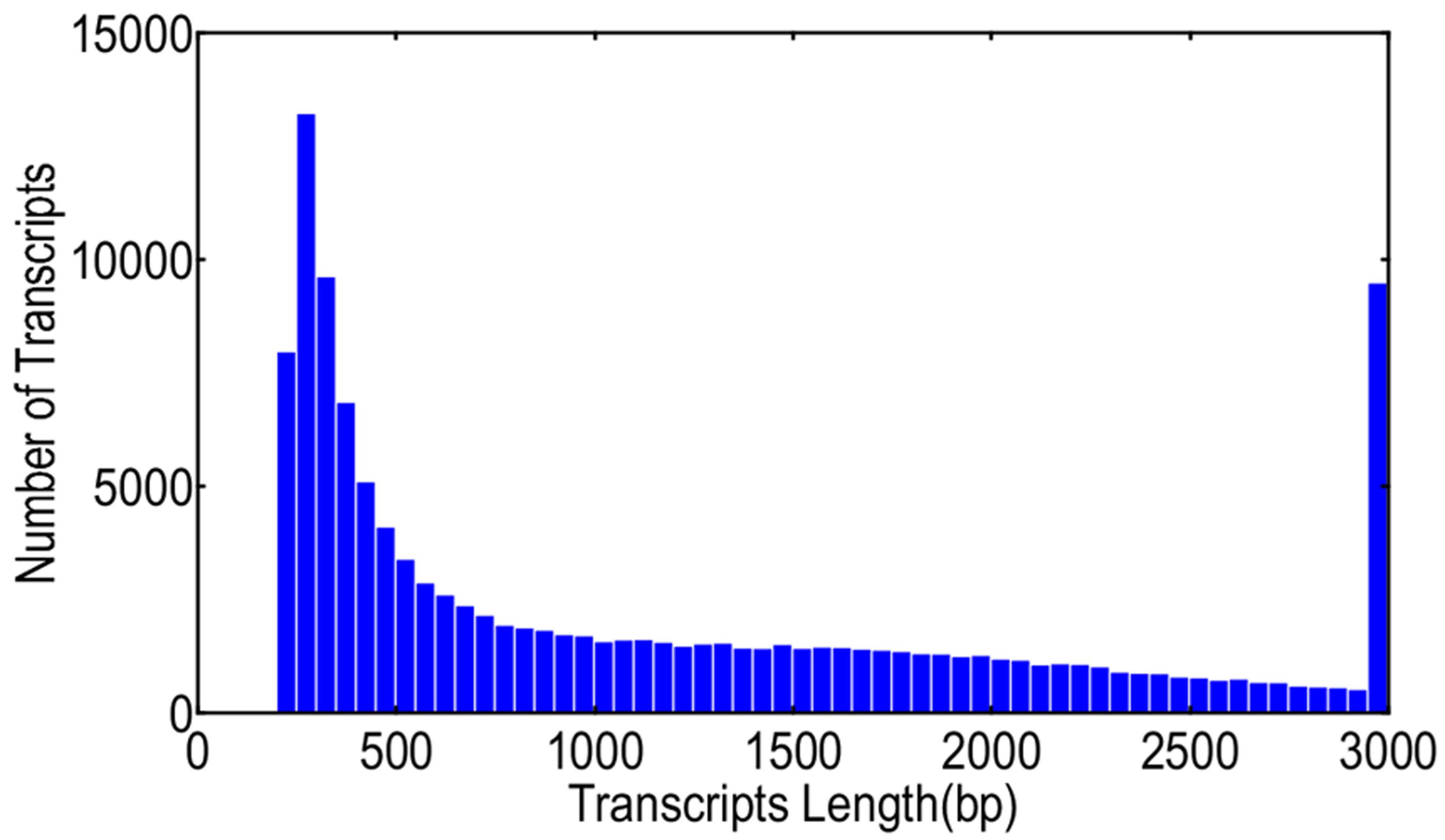

Supplement: Figure S2 — Statistics of assembly length for transcripts. [file Image2.JPEG]

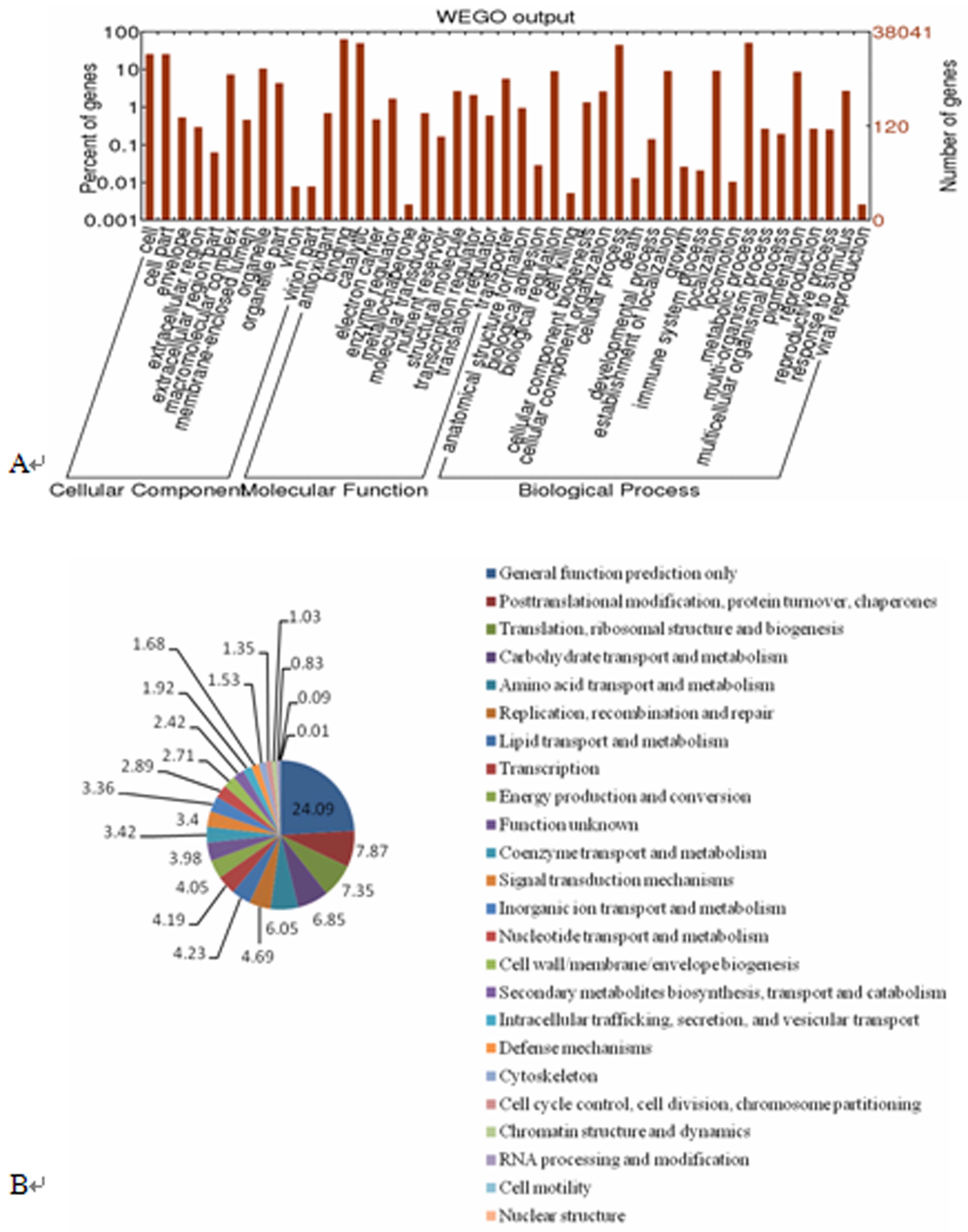

Supplement: Figure S3 — GO and COG classification of transcripts. (A), GO categories of transcripts; (B), COG classification of transcripts. [file Image3.JPEG]
