# Supplementary material for: Transcriptomic Analysis Reveals Key Genes Related to Betalain Biosynthesis in Pulp Coloration of Hylocereus polyrhizus
Source: Front Plant Sci. 2016 Jan 5;6:1179. doi: 10.3389/fpls.2015.01179 (PMC4700300; doi:10.3389/fpls.2015.01179)
Supplement: Table S1 — Primers used in this study. [file Table1.DOC]

| **Primer name** | **Forward primer sequence (5′to 3′)** | **Reverse primer sequence (5′to 3′)** |
| --- | --- | --- |
| actin | CTTCCATACCAATGAATGAGG | AACCGCCAAGAGTAGTTCTG |
| comp16058_c0_seq1 | GCTCCAGCCGAACCATACCC | TCTTCCTAAAACTCCGCCAT |
| comp37375_c0_seq2 | CCTGCTAATGACCCTCGTA | GCTATCCCAGTTCCAAAAAG |
| comp37674_c0_seq1 | GAGTCAAAGGTGTTTTATCT | TTATCGGGTGAGTAGGAGGA |
| comp36993_c0_seq3 | GCGGGAACAGATACAACTTC | CCTTGATTGACCTTCGCCTA |
| comp36993_c0_seq4 | TCCTCATTTTCATCCTTCGT | ATTTCTTTGGCTACATCGGG |
| comp30986_c0_seq1 | GGTCGCCGATGCTTTCTGTT | TTCTTGTGCTCGCTTTGCCA |
| comp32889_c0_seq1 | AATGTTGTTGTGGATAGGTT | AGACGGCTGGGATTTTGTTA |
| comp37692_c0_seq1 | TGTGCCTTCTGTATCTGCCG | TGTCTTGAACCCTCCTGCTA |
| comp32369_c0_seq2 | GTTTCATTGGGTGCTGTCAT | CTTGGGTCTATCCCTTGGTA |
| comp35191_c2_seq2 | CGCCTTCACAACTTCACTTA | TCCCAATCTCCACCATTATC |
| comp29696_c0_seq1 | GACTGATGTGCCTGCTGTTA | AGTTCCTCTACCCTCCTTGC |
| comp36238_c0_seq2 | CATTCCATTTCCAGCACAAG | GGAGTTAGTTGAGGGAGGTTGA |
| comp37261_c1_seq14 | CGCAGAACAACTGAGGGAGT | GGGTGAGTAGGTGGATTGGTT |
| comp26435_c0_seq1 | TGGCTCCTGTGTTACCTTGA | AATCCACCACCGAGTTTGAC |
| comp28147_c0_seq1 | GAAGAGCAGGGAGTCTACGA | GGAACAACAACAACCCAGA |
| comp33513_c0_seq1 | GTGTGGACCTGACTGTTGA | CATTGTTGGACTTTGTGTGTG |
| comp32700_c0_seq1 | ACGATGAGGGTATGATGGAT | TCTTGTTCTGCTCCGACTG |
| comp34286_c2_seq1 | GGTTCGGGTGTTCAGTATTC | CCCTCCATTCAAACTATCTTTC |
| comp30867_c0_seq1 | CACCACCACCACCATCTA | TGATAAACTGGGTCTCTTCCA |
| comp26707_c0_seq1 | GGTCTGATTATGTGGTCGTG | AGAGTGAAGTTGGGATGAGC |
| comp28147_c0_seq1 | CTTTCTTGCTCTTTCCCTTC | GTCCCTGATTGGTTCACATT |
| comp37672_c0_seq1 | ACGGCAACACCCAACTATT | CGTCTCTCATCGTCACTTCT |
| comp37717_c0_seq1 | AGGGCAACACCAGTAAAGAG | TCACAGCATCAGCAAGGAT |
| comp23614_c0_seq1 | ATCAGAAGGCGACAAAGC | GCACATCAATAGGGAATGGA |

Table S1 Primers used in this study
