# Supplementary material for: Transcriptomic Analysis Reveals Key Genes Related to Betalain Biosynthesis in Pulp Coloration of Hylocereus polyrhizus
Source: Front Plant Sci. 2016 Jan 5;6:1179. doi: 10.3389/fpls.2015.01179 (PMC4700300; doi:10.3389/fpls.2015.01179)
Supplement: Table S2 — Expression statistics of transcripts between the white and red pulp libraries. [file Table2.DOC]

**Table S2 Expression statistics of transcripts between the white and red pulp libraries**

| Class | Number(#) of Transcripts | % |
| --- | --- | --- |
| Total transcripts | 122677 |  |
| Expressed transcripts | 122668 | 99.99 |
| Expressed in red stage | 117185 | 95.53 |
| Expressed in white stage | 116582 | 95.04 |
| Expressed both in white and red stages | 111099 | 90.57 |
| Expressed only in red stage | 6086 | 4.96 |
| Expressed only in white stage | 5483 | 4.47 |
| Differentially expressed transcripts  (p≤0.01 && (ratio≥2 or ratio≤0.5) | Total # | 8871 |
|  | Up # | 4107 |
|  | Down # | 4764 |
